# Potential Molecular Targeted Therapy for Unresectable Hepatocellular Carcinoma

**DOI:** 10.3390/curroncol30020105

**Published:** 2023-01-18

**Authors:** Shashank Kumar, Abhay Kumar Pandey

**Affiliations:** 1 Molecular Signaling & Drug Discovery Laboratory, Department of Biochemistry, Central University of Punjab, Guddha, Bathinda 151401, Punjab, India; 2 Department of Biochemistry, University of Allahabad, University Road, Prayagraj 211002, Uttar Pradesh, India

**Keywords:** liver cancer, survival, recurrence, clinical trial, therapeutic target

## Abstract

Hepatocellular carcinoma (HCC) is one of the most prevalent and lethal cancers, representing a serious worldwide health concern. The recurrence incidence of hepatocellular carcinoma (HCC) following surgery or ablation is as high as 70%. Thus, the clinical applicability of standard surgery and other locoregional therapy to improve the outcomes of advanced HCC is restricted and far from ideal. The registered trials did not identify a treatment that prolonged recurrence-free survival, the primary outcome of the majority of research. Several investigator-initiated trials have demonstrated that various treatments extend patients’ recurrence-free or overall survival after curative therapies. In the past decade, targeted therapy has made significant strides in the treatment of advanced HCC. These targeted medicines produce antitumour effects via specific signals, such as anti-angiogenesis or advancement of the cell cycle. As a typical systemic treatment option, it significantly improves the prognosis of this fatal disease. In addition, the combination of targeted therapy with an immune checkpoint inhibitor is redefining the paradigm of advanced HCC treatment. In this review, we focused on the role of approved targeted medicines and potential therapeutic targets in unresectable HCC.

## 1. Introduction

The Global Burden of Disease places liver cancer as the second leading cause years lost due to ill health. In the past 40 years, the incidence of liver cancer has risen drastically with increased mortality. Hepatocellular carcinoma (HCC) is the most common liver cancer subtype, produced from liver cells or intrahepatic biliary epithelial cells [1,2]. About 90% of initial liver tumours are HCC. One of the main curative methods for HCC is liver resection. After surgical resection, the annual recurrence rate of HCC is 10% and increases to 70–80% after 5 years [3]. Identification of patients with a high risk of HCC recurrence following curative surgical resection is crucial from a therapeutic standpoint. The five-year survival rate following curative resection is commonly stated to be between 40 and 70 percent. Up to 50% of individuals with resectable HCC who first have surgery later experience another recurrence [4]. The high risk of recurrence has been ascribed to the lack of efficient adjuvant therapy as well as the presence of occult micrometastasis from the primary tumour at the time of surgery. Tumour size, tumour count, and portal vein invasion are additional factors that are linked to early recurrence following resection. However, a pathological assessment for an early HCC recurrence has not been provided [4,5]. In this review, we look at current developments in targeted therapy to combat HCC recurrence and the underlying mechanism. We also give an outline of the key areas of HCC research, such as cutting-edge clinical trials that promise to enable significant advancement over the course of the following ten years. Various target-specific drugs and their mechanisms of action are described in the subsequent section of the review.

## 2. Targeted Therapy for Unresectable HCC

### 2.1. Belinostat: A Histone Deacetylase Inhibitor (HDACi)

Histone deacetylase (HDAC) enzymes are mostly zinc-dependent proteins that compress chromatin and restrict the transcription of related genes by catalysing deacetylation of histone proteins. Increased HDAC expression is associated with cancer initiation/progression and thus, it is an appropriate therapeutic target [6]. HDAC inhibitors (HDACi) are now acknowledged as a distinct and powerful class of anticancer drugs. In HCC cell lines and xenografts, it has been shown that inhibitors of HDAC cause apoptosis and tumour regression in addition to having anti-proliferative, anti-metastatic, and anti-invasive properties [7]. One member of the family, belinostat, has undergone testing for the treatment of hepatocellular carcinoma both alone and in combination with other chemotherapies and biological agents [8]. PXD101 (belinostat), an inhibitor of histone deacetylase, inhibits the development of hepatocarcinoma cells in a dose-dependent manner and causes histone acetylation. PXD101 therapy induces apoptosis without having a large impact on viral gene expression. Twelve cellular genes with tumour suppressor roles experienced varied changes in expression after being exposed to PXD101 for up to 48 h [9]. Phase I and phase II trials consisted of 19 and 36 patients, respectively. In phase II trial, the median progression-free survival (PFS) and OS were 2.83 and 8.89 months, respectively. The study concluded that further in-depth studies are required, as the targeted PFS endpoint was not achieved [10]. Wang et al. (2013) studied pharmacokinetics of belinostat in HCC patients to determine its associated metabolic mode of action. The study identified UGT1A1-mediated glucuronidation as a major mode of metabolic pathway alteration in HCC patients. The clinical significance of this finding remains to be determined [11].

### 2.2. Bevacizumab: A Vascular Endothelial Growth Factor (VEGF) Inhibitor

The vast majority of HCC cases are diagnosed at an advanced stage, despite the fact that many patients with the condition present at a younger age. With surgery or local ablation, patients who present with early- and intermediate-stage illness may be cured. The interaction between tumour cells and the elements of the tumour microenvironment is evidently suggested to be a significant contributor to the development of HCC. The establishment of an immunosuppressive environment is a crucial stage in the development of tumours, and as HCC is an inflammation-associated tumour, targeting the immune network may be a useful treatment strategy. Previous studies have shown that in unresectable HCC, the simultaneous blockage of vascular endothelial growth factor inhibitor (VEGF) and PD-L1 results in a longer progression-free survival [12]. Philip et al. (2012) studied the effect of erlotinib and bevacizumab as combination therapy in HCC patients in a two-stage phase II clinical trial. The combination therapy resulted in minimal activity with a median survival of 9.5 months. Further studies are required to understand the effects of combination therapy in molecularly categorised patients [13].

Metastatic recurrences after curative hepatic resections are common; thus, therapeutic interventions to produce antiangiogenesis effects have been attempted to aid HCC prognosis. VEGF, a dimeric glycoprotein and an important angiogenic factor, increases blood vessels’ permeability and is a potent mitogenic agent. Thus, by increasing angiogenesis and metastasis, VEGF is directly involved in HCC recurrence. Niu et al. (2000) showed higher serum levels of VEGF in the increasing order of normal, cirrhosis, and HCC patients [14]. In a different study, Guo et al. (2012) found significantly higher serum VEGF levels in HCC patients compared to normal individuals. Different studies correlated higher serum VEGF levels with poor overall survival in HCC patients, indicating VEGF’s prognostic value in the disease [15,16].

Bevacizumab, a humanised monoclonal antibody targeting VEGF-A, has shown anticancer efficacy in different types of cancers. The antibody inhibits the interaction between VEGF and its receptor molecule by directly binding to it, which in turn checks the growth and angiogenesis in tumour cells [17]. Bevacizumab showed better efficacy in HCC patients compared to available standard therapy and has been approved (2020) as an initial therapy for HCC patients with metastasis or those who cannot be treated with surgery [18].

### 2.3. Bortezomib: A Proteasome Inhibitor (PI)

Proteasome inhibitors (PI) have shown promising potential as chemotherapeutic agents in the treatment of HCC [19,20]. Dawson reviewed the mechanisms of different PIs in HCC [21]. As the first in class, bortezomib (BZB) is recommended for the treatment of mantle cell lymphoma and refractory multiple myeloma. It was given FDA approval in 2003 and is now the only PI with clinical approval [22]. The use of BZB alone or in combination with other treatments for HCC has drawn a lot of attention. The role of BZB in the management of non-surgical and metastatic HCC was expertly examined by Huang et al. (2018) as an alternative or a supplement to the current treatment paradigm [23]. Ciombor et al. (2014) studied the effects and tolerability of BZB and doxorubicin (DOX) in HCC patients in a phase II clinical trial [24]. The proteasome inhibition was correlated with the therapy response and survival in advanced HCC patients. The combination therapy showed suitable tolerance in the patients, but the primary endpoint (objective response rate) was not met. The study suggested the prognostic role of proteasome inhibition markers in advanced HCC patients [24].

### 2.4. Cixutumumab: An IGF-IR Signalling Inhibitor

The insulin-like growth factor (IGF) pathway includes a family of receptors and ligands. Since the IGF-1R (insulin-like growth factor-1 receptor) route regulates cellular motility, apoptosis, and proliferation, it has been suggested that signalling through this pathway contributes to a variety of cancers [25]. In HCC, IGF-IR expression and activity are both increased. The genesis and progression of HCC tumours are also hypothesised to be accelerated by elevated IGF expression [26]. HCC cell lines have experienced growth suppression and apoptosis as a result of pre-clinical investigations with a range of anti-IGF-1R techniques. In addition, studies indicate that the IGF-IR signalling cascade has an angiogenic component since it can trigger the release of VEGF [27]. As a result, anti-IGFR medication increases the efficacy of anti-VEGF therapy by further reducing tumour-associated VEGF release. Recombinant human IgG1 monoclonal antibody cixutumumab (formerly IMC-A12, ImClone Systems, Inc, Bridgewater, NJ, USA) is aimed at IGF-1R. IGF-1R is internalised and degraded by cixutumumab, which also potentially binds to it (Kd = 0.04 nM). Cixutumumab also inhibits the interaction between IGF and their respective receptors and thereby reduces cell viability/proliferation in HCC cells [28,29,30]. Fully human IgG1 monoclonal antibody cixutumumab binds to IGF-R1 with great specificity. The IFG-R1 tyrosine kinase domain is activated by IGF-1. The Akt survival route and the MAP kinase proliferation pathway are both activated by phosphotyrosine residues. In a human tumour xenograft model, cixutumumab efficiently prevented ligand-induced phosphorylation, which inhibits tumour cell proliferation and induces apoptosis. Through internalisation and degradation of the receptor, cixutumumab regulates the receptor localisation on tumour cell surface [31]. A phase II trial (NCT00639509) studied the efficacy of cixutumumab in advanced liver cancer. The study showed an 8-month median overall survival in patients treated with cixutumumab.

### 2.5. Doxorubicin: A Topoisomerase I and II Inhibitor

DNA topoisomerase I and II induce DNA breaks. Liu et al. (2003) showed significantly increased expression of TOP I in HCC tissue samples compared to adjacent normal tissue [32]. It has been shown that TOP2 induces cell invasion and EMT in HCC cells, which indicates its candidacy as a therapeutic target [33].

The stage of the disease determines the recommended course of HCC treatment. Transarterial chemoembolisation (TACE) is advised for HCC patients who are unresectable and at the intermediate stage. TACE entails delivering the cytostatic agent(s) to the tumour through the hepatic artery using a drug delivery system (DDS). The DDS causes embolisation, which impairs blood flow to the tumour, causes hypoxia, increases drug concentrations, and prolongs drug residence periods in the tumour target region. The predicted median survival is increased by about 4 months with the use of palliative TACE treatments [34]. Cytostatic drugs placed into drug-eluting beads or emulsified in Lipiodol^®^ (LIP) are two popular DDSs utilised for TACE therapy for intermediate-stage HCC. Chemotherapeutic chemicals that have a positive charge can be placed into DCB (DC Bead^®^) for later local release [35]. The primary cytotoxic agent for intermediate-stage HCC is doxorubicin (DOX) used as its hydrochloric salt. Anthracycline, antibacterial, and antineoplastic medication DOX is used off-label to treat intermediate HCC despite having various cancer indications [36]. At least three anticancer mechanisms, including intercalation to DNA base pairs, free radical production, and reversible binding to topoisomerase (TOP) I and II, appear to be responsible for the pharmacological actions of DOX [36]. The amount of DOX needed to inhibit growth by 50% (IC_50_) in vitro depends on both the period and the cell type [37]. Gish et al. (2007) compared the efficacy of DOX and nolatrexed in advanced HCC patients in terms of overall survival in a phase III trial. Dox showed lesser toxicity and increased survival in HCC patients compared to nolatrexed-treated patients [38].

### 2.6. Erlotinib: An Epidermal Growth Factor Receptor (EGFR) Inhibitor

EGFR has attracted a lot of interest as a potential therapeutic target of tumour cells. As a key player in signal transduction pathways involved in different cancer hallmarks, EGFR is typically overexpressed in solid tumours. In some tumour types, its overexpression is associated with disease progression and a worse prognosis [39,40]. Erlotinib’s main therapeutic target, EGFR, possesses a transmembrane segment, a cytoplasmatic tyrosine kinase domain, and an extracellular cysteine-rich ligand-binding region, which serve as the binding sites for kinase inhibitors such as erlotinib. Through conformational changes and subsequent phosphorylation of tyrosine residues, extracellular ligand binding initiates events such as active receptor homo/hetero dimerisation and the formation of binding sites for subsequent signal transducers, initiating a series of events that lead to the development and growth of tumours [41]. In HCC, the EGFR is typically overexpressed. The increase in ligand–receptor contact rather than point mutations or amplifications might be the mechanism through which EGFR signalling in HCC gains function. Apoptosis and cell cycle-regulating genes express differently as a result of erlotinib treatment for HCC [42].

The tyrosine kinase inhibitor drug erlotinib inhibits epidermal growth factor receptor (EGFR) and is orally accessible. Erlotinib has the ability to prevent tumour cell growth, invasion, metastasis, and angiogenesis [43,44]. Thirty eight patients with metastatic or unresectable HCC participated in a phase II study to assess erlotinib’s effectiveness. The most common grade 3 to 4 toxicities were fatigue (8%) and diarrhoea (8%). Child–Pugh classification and toxicity severity (grade 3 or higher) were correlated; only 22% of Child–Pugh A patients and 70% of Child–Pugh B patients had severe toxicity (*p* = 0.02). After 24 weeks, 32% of the patients were no longer progressing. Only 9% of responses were confirmed in total [45]. Erlotinib alone appears to have relatively little effectiveness against HCC, and more randomised studies are required to assess the drug’s potential advantages for HCC patients [42]. Erlotinib has been tested in few clinical trials for the treatment of advanced HCC, but due to a lack of detailed analysis, the mode of erlotinib efficacy was not clear in advanced HCC. Thus, for a complete picture of the safety and effectiveness of erlotinib, Zhang et al. (2016) carried out a comprehensive literature review and concluded that erlotinib should be considered as a potential therapeutic option [46].

### 2.7. Galunisertib: A TGF-β Pathway Inhibitor

TGF-β is a multifunctional cytokine that regulates a variety of cellular processes in most cells, including proliferation, cellular differentiation, adhesion, migration, and apoptosis. TGF-β serves as a tumour suppressor in normal tissues, but as tumour cells develop strategies to circumvent its actions, it promotes the growth of the tumour. Although the transition from a tumour suppressor to an oncogenic state is not fully understood yet, intrinsic and extrinsic variables appear to be significant [46,47]. A mesenchymal phenotype, the loss of cell polarity, and the development of motile characteristics during epithelial–mesenchymal transitions (EMT) are thought to be crucial intrinsic changes in the tumour cells. Extrinsic variables from the tumour microenvironment, such as angiogenesis, inflammation, and fibroblast activation, also contribute to TGF-β signalling’s pro-tumourigenic potential. Modifications to the TGF-β signalling system can potentially contribute to tumour growth in addition to changes in the tumour tissue. Therefore, TGF-β plays a crucial role in the molecular aetiology of HCC; targeting TGF-β could offer new therapeutic insights [48,49]. Three of the several small-molecule inhibitors of TGF-RI/II/ALK5 that have been created are currently being tested in human clinical trials. The sole TGF-β pathway inhibitor undergoing clinical testing in HCC patients is galunisertib (LY2157299), a selective ATP mimetic inhibitor of TGF-RI/ALK5 (NCT01246986). Galunisertib has recently been demonstrated to effectively block the expression of p-Smad2 and invasion in three HCC models in vitro, but not proliferation [50].

TGF-β, a pro-inflammatory/profibrotic cytokine, is a complimentary signalling molecule to VEGF that aids in the advancement of various HCCs. A subgroup of HCCs has higher levels of TGF-β, which increases neovascularisation and encourages immune evasion and immunosuppression, as well as migration and invasion [51]. In fact, compared to HCCs not controlled by TGF-β, those showing indications of TGF-β activation also behave more aggressively and suggest a worse prognosis [48,52]. The oral TGF receptor I (ALK5) small molecule inhibitor galunisertib disrupts TGF signalling and harms HCC both in vitro and in vivo [49]. The treatment slows the growth of HCC, lessens tumour vascularity, lowers HCC motility and invasiveness, treats fibrosis, and boosts the local immune response. Galunisertib monotherapy has been shown in phase I and II studies to have a positive safety profile that does not overlap with that of antiangiogenic drugs and to provide a small amount of therapeutic benefit via cytostatic disease control in patients with advanced HCC [50,53]. A phase Ib/II study was designed (NCT02423343) to check the efficacy of galunisertib dose escalation and its safety and tolerability in HCC patients. The study was terminated in its early stage due to the low enrolment of HCC patients.

### 2.8. Nivolumab: A Programmed Cell Death Protein 1 (PD-1) Inhibitor

PD-1 (a coinhibitory receptor) and its ligand (PD-L1)-mediated pathway have been implicated in compromised tumour immunity. The use of anti-PD-L1 antibodies enhances tumour sensitivity and decreases tumour growth [54]. Wu et al. (2009) showed that the interaction of PD-1 with its ligand contributes to immune suppression in human HCC. The study suggested that PD-1 and PD-L1 interaction blockade carry important therapeutic implications in HCC [55]. Scheiner et al. (2019) studied the safety and efficacy of nivolumab in combination with other antibodies in a cohort of HCC patients. The study indicated the better efficacy and safety profile of nivolumab in HCC patients [56]. In a meta-analysis, the higher expression of membrane-bound and soluble PD-1 was positively correlated with shorter overall survival in HCC patients [57]. Large important clinical trials have effectively demonstrated the notion of inhibiting PD-1 activity in cancer patients.

Human immunoglobulin G4 (IgG4) monoclonal antibody nivolumab was created by Bristol-Myers Squibb (BMS) to bind to the PD-1 receptor. The clinical success of the antibody is due to its variable region’s high affinity (Kd 3.06 pM) and specificity (no binding to CD28, ICOS, or CTLA-4) in binding the target (interaction with the PD-1 N-loop). Nivolumab has high affinity and specificity for the PD-1 epitope that it targets [58,59]. A phase I/II clinical trial focused on HCC assessed the efficacy of nivolumab, a completely human immunoglobulin G4 monoclonal antibody to programmed cell death protein 1 (PD-1). Nivolumab showed a controllable safety profile throughout dosage escalation, including acceptable tolerability. Regardless of the aetiology, PD-L1 expression in the tumour, or previous sorafenib exposure, responses were seen. In patients with Child–Pugh B cirrhosis, nivolumab showed activity and tolerability with ORR and DCR of 10% and 55%, respectively. Nivolumab was given conditional FDA clearance based on this trial (NCT02576509) [60].

Nivolumab and pembrolizumab were evaluated for safety and efficacy in patients with advanced HCC who had previously failed to respond to sorafenib treatment in the CheckMate-040 and KEYNOTE-224 studies, which provided the foundation for the FDA’s accelerated approval of these drugs as second-line therapies [61,62]. Nivolumab’s safety and effectiveness in an Asian cohort were confirmed by a subanalysis of the data from CheckMate-040 [63]. An outstanding response to anti-PD-1 therapy has also been recorded in a case report, and ICIs have demonstrated potential efficacy and tolerability in advanced HCCs as systemic first-, second-, third-, and fourth-line treatments, with median overall survival (OS) and progression-free survival (PFS) of 11.0 and 4.6 months, respectively [56,64]. Although the phase III KEYNOTE-240 trial’s results for improved PFS and OS did not reach the pre-specified statistical significance, they were consistent with those of KEYNOTE-224 [65]. Pembrolizumab’s role in cases of advanced HCC with a viral background may be clarified by the KEYNOTE-394 study, which is now being conducted with Asian patients. Nivolumab did extend OS regardless of the PD-L1 expression profile in HCC patients and also increased the survival of HCC patients with HBV/HCV as the aetiology and prevented the reactivation of hepatitis. An anti-PD-1 inhibitor from China called camrelizumab (SHR-1210, Hengrui Pharmaceutical, Jiangsu Lianyungang, China) is being tested for the treatment of Hodgkin lymphoma and HCC. In a multi-centre, open-label, parallel-group, randomised, phase II trial (NCT02989922), it was discovered to have antitumour activity in Chinese patients with advanced HCC who had previously undergone treatment. This finding supports the efficacy of PD-1 therapy for Chinese patients with HBV-related HCC [66]. It is hoped that the outcomes of additional trials testing novel ICIs such as durvalumab, avelumab, tislelizumab, sintilimab, tremelimumab, ipilimumab, spartalizumab, and toripalimab will be encouraging and provide additional treatment options for HCC patients, especially those who have relapsed on first-line therapies. Dual ICI therapy and ICI combination therapy are two further strategies being used to improve the effectiveness of ICIs as therapeutic agents. CheckMate 9DW’s early outcomes for dual ICI treatment were astounding: the objective response rate was 32%, which was higher than monotherapy of any ICIs alone. Using the combination of the therapeutic agents (such as tivantinib and capmatinib), anti-PD1 and anti-PDL1 created an additive effect that inhibits the formation of HCCs in mice. MET-mediated phosphorylation results in a lower expression of PD-L1 [67]. For the treatment of advanced HCC, individual case studies have also shown encouraging outcomes in TKI and anti-PD1/PD-L1 agents combination therapy [68,69,70]. Early phase data that showed promise prompted the development of many immune checkpoint inhibitor-based combination treatment regimens, the outcomes of which will be made public in the coming years [71]. Several immune checkpoint inhibitor-based combination treatment approaches have been started as a result of encouraging early-phase data; the outcomes of these approaches will be known in the coming years. Additionally capable of expressing PD-L1, tumour cells can do so while eluding the immune system [72,73].

### 2.9. Peretinoin: A Cellular Retinoic Acid-Binding Protein

Despite effective treatment, HCC has a high risk of coming back. Patients with HCC brought on by HBV or HCV experience the same mechanism of recurrence. Additionally, although it might be challenging to pinpoint the mode of recurrence of certain lesions, the time of recurrence is thought to vary. For instance, 2 years after the initial tumour has had radical treatment, intrahepatic metastasis recurrence is more common than metachronous multicentric recurrence. The prognosis of patients with HCC be enhanced by actively reducing HCC recurrence in addition to early detection and therapy [74,75]. Kanemastu et al. (1988) studied the level of cellular retinol binding protein levels in HCC tumour samples compared to adjacent normal parenchymal tissue and found no significant difference between the two groups [76]. In a different study, Schmitt-Gräff et al. (2003) reported differential CRBP-1 levels in cancerous and normal liver cells. The study revealed that modulating the CRBP-1 expression in HCC might inhibit the growth and progression of tumours [77]. Lee et al. (2010) showed that targeting CRBP induces apoptosis in HCC cells [78]. Recently, Liu et al. (2021) found that CRBP-1 is a crucial player in the initiation and progression of HCC, which provides an independent prognostic biomarker and therapeutic target for the diagnosis and treatment of HCC [79,80].

Peretinoin is a synthetic polyprenoic acid with retinoid-like characteristics that binds to cellular retinoic acid-binding protein. Peretinoin’s method of action involves the activation of target genes’ transcription through the receptors (such as retinoic acid) and other transcriptional complexes. In HCC cells, peretinoin was discovered to alter the expression of genes that control cellular differentiation, cellular proliferation, and apoptosis. According to several pharmacologic studies, peretinoin prevents the growth of subclinical cancers and/or the carcinogenesis of precancerous lesions in the liver, thus preventing the recurrence of HCC [81,82,83].

Initially, peretinoin was used to treat skin disease. Later on, a clinical study in curative resected HCC patients on therapy showed increased survival rates and low recurrence of the disease. Based on these encouraging outcomes, peretinoin was created in 1997 under the trade name NIK 333 (Kowa Company, Ltd., Chuo-ku, Tokyo), and clinical trials on the substance began in February 2012 under the new designation K-333 [84,85]. A phase III trial study (NCT01640808) checked the efficacy of peretinoin (600 mg oral dose twice a day) in terms of recurrence-free survival in HCC patients as a primary measure. The disease-free survival and time to recurrence were studied as secondary outcomes in HCC patients. Increased recurrence-free survival was observed in the peretinoin-treated group compared to the placebo arm. Similarly increased disease-free survival and time to recurrence were also observed in the peretinoin-treated arm. Extensive pre-registration phase III placebo-controlled trials using peretinoin are now being conducted in Asia to assess its effectiveness and safety in patients with virus-associated HCC after total tumour excision (Table 1) in light of new information [86].

### 2.10. Selumetinib: A Mitogen-Activated Protein (MAP) Kinase Inhibitor

There is evidence that the RAF/MEK/ERK pathway is important in the development of HCC. It has been shown that in 50 to 100 percent of human HCCs, this route is activated [78,87,88,89]. This is mostly attributed to receptor tyrosine kinases, such as IGF-1, EGF, or c-MET, which are involved in autocrine/paracrine signalling. Furthermore, it was recently discovered that HCCs seem to express fewer RAS pathway inhibitors than normal tissues. Mixed results have been seen in studies of MEK/ERK inhibition in HCC during in vitro and in vivo experiments [90]. Klein et al. used a variety of techniques to block the MEK/ERK pathway, and they found that several HCC cell lines had decreased proliferation and increased apoptosis [91]. Selumetinib was used by Huynh et al. against HCC cell lines, and they once more showed that it was active in HCC experimental models. Selumetinib (AZD6244, ARRY-142886) is a competitive inhibitor of MEK1/2, and its recommended oral phase II dose has previously been determined to be 100 mg twice daily [92,93].

Selumetinib was evaluated as a first-line therapy in a recent phase II study for individuals with advanced HCC. Nineteen patients were enrolled in the first stage, with plans to recruit 25 more in the second stage if at least one objective response (the primary end point) had been seen. This means that a total of 44 patients could have participated in the research. The trial was terminated at the interim analysis because no radiographic response was seen in the study population. Three (27%) of the eleven patients with increased α-fetoprotein showed reductions of 50% or more, and the median time to progression (TTP) was 8 weeks. The level of toxicity was consistent with selumetinib toxicity studies in patients without cirrhosis. Selumetinib appeared to have a negligible single-agent effect, despite the pharmacokinetic analysis showing that target activation was suppressed [94]. A phase II trial study (NCT00604721) checked the efficacy of selumetinib in locally advanced or metastatic liver cancer in terms of objective response rate as the primary measure. The median progression-free and overall survival were studied as secondary outcomes in the patients. The study reported moderate liver toxicity with PFS and OS of 1.4 and 4.2 months, respectively.

### 2.11. Sofosbuvir: A Nucleotide Analogue

A non-structural protein 5B (NS5B) inhibitor, sofosbuvir (SOF) is also known as PSI-7977. It received Fast Track FDA approval in August 2010. Two molecules with a similar chemical makeup, PSI-7976 and PSI-7977, are combined in the medication. Both PSI-7976 and PSI-7977 molecules quickly transform into the same active triphosphate when the medication enters the liver cell. The first ribonucleoside analogue inhibitor, SOF, was authorised in 2013 for the treatment of chronic hepatitis C. The FDA approved the medicine in 2014 as a prodrug and nucleotide analogue [95,96]. Widely used DAAs for treating HCV, including SOF, specifically target the HCV RNA-dependent RNA polymerase (RdRp). Importantly, SOF-based regimens were used to treat the majority of reported cases of HCC development. In a Huh7-based subgenomic replicon, SOF effectively reduced HCV replication, as predicted. By using MTT and Ala blue tests, SOF therapy for 48 or 72 h had no discernible impact on the development of the majority of HCC cells. Surprisingly, however, SOF improved all four HCC cell lines’ single cell-based clonogenic potential. The much higher quantity and size of established colonies justify this aspect. Contrarily, placebo therapy has no such impact. Thus, it has been unequivocally shown that SOF has a direct stimulating influence on the beginning and expansion of clonogenic processes based on single HCC cells, but not on the expansion of the majority of HCC cells. The tumour microenvironment is a challenging and developing field, so neither these results nor the previous study should rule out the possibility of a secondary effect of DAAs on initiating the development of HCC. Therefore, future experimental and clinical research must work together to shed light on this worrying situation and provide a mechanistic understanding [97]. Atif et al. (2022) recently came to the same conclusion that SOF neither causes nor eradicates HCC based on the expression patterns of a few chosen genes in SOF-treated HCC cell lines [98].

HCV is a positive strand virus that contains a single stranded (ss) RNA genome, encoding a polyprotein that is processed into several structural and non-structural proteins. The NS5B possesses RdRp activity, catalyses HCV RNA replication, and is a prime target for antiviral drug discovery. Nucleoside/nucleotide analogs and non-nucleoside inhibitors are the two important groups of HCV RdRp inhibitors. An RdRp-activity-targeting-drug binds to its allosteric site and inhibits the imitation step by altering the structure–function relation of the protein [99]. HCV-induced HCC is a complex process which includes the development of a pro-carcinogenic environment (cirrhosis) and promotes malignancy in the presence of viral proteins. This gradual process can take up to four decades to develop, during which cell cycle-related proteins become mutated and ultimately convert normal hepatocytes into malignant types [100].

HCC development is known to be correlated with chronic hepatitis C virus (HCV) infection. Although the number of new HCV cases has decreased, people with HCC are more likely to have HCV infection. Direct-acting antiviral (DAA) therapies have revolutionised the management of HCV infection. Compensated DAA agents achieve sustained virologic response (SVR) in the liver function, and reduced need for liver transplantation was also achieved [101]. Mixed responses have been reported concerning the outcome of DAA therapy. Contrary to expectations, some recent studies found an unexpectedly high rate of HCC formation following DAA treatment, while other research found no such risk. Due to the diverse demographics and methodology used in many studies, it is still difficult to draw a firm conclusion on this matter.

### 2.12. Temsirolimus: An mTOR Pathway Inhibitor

The significance of the mammalian or mechanistic target of rapamycin (mTOR) signalling pathway in controlling liver cancer cell growth was revealed by Li et al. (2013) [102]. The study team demonstrated that temsirolimus suppresses the activity of mTOR and its downstream components, which has inhibitory effects on Bel-7402 liver cancer cells. They proposed that mTOR inhibition might be used as a treatment for liver cancer [102]. Among the several molecular pathways involved in HCC development, mTOR is one of the most investigated. A conserved checkpoint serine/threonine kinase called mTOR takes part in a number of molecular signalling pathways both inside and outside of cells. The downstream effects of mTOR activation include the response to cellular hypoxia and control over intracellular energy storage, growth, and replication. Since mTOR is associated with cellular growth and replication, inhibiting mTOR may be crucial in preventing the development of cancer. The rapamycin analogues known as mTOR inhibitors are able to deactivate mTOR by attaching to its C-terminal kinase domain. The three inhibitors that are most frequently used in clinical settings are sirolimus, everolimus, and temsirolimus. Since mTOR inhibitors are also immunosuppressive drugs, their use in liver transplant patients who have previously had HCC have significant therapeutic implications for preventing tumour recurrence. Because of the impaired liver function, standard chemotherapy drugs are ineffective against HCC and poorly tolerated [103,104,105]. Everolimus (RAD001), temsirolimus (CCI-779), and deforolimus are three examples of rapalogs whose bioavailability has been increased due to the early success of rapamycin (AP23573). The primary focus switched to anti-cancer therapy as a result of the significant function of mTOR in cell growth and metabolism, and temsirolimus (CCI-779) was licenced for the treatment of renal cell carcinoma in 2007. The first-line therapy portion of its trial in advanced HCC patients (NCT01079767) was stopped due to its toxic effects [106]. Knox et al. (2014) studied the efficacy of bevacizumab and temsirolimus combination therapy in advanced HCC patients. The study demonstrated an increased overall response rate (ORR) with PFS and OS [107].

## 3. Combination Targeted Therapy—A Way out to HCC Recurrence?

The United States Food and Drug Administration (FDA) approved the multi-kinase inhibitor sorafenib as a systemic treatment for HCC on the basis of a phase III trial that showed non-surgical candidates with intact liver function with better overall survival over placebo [108,109]. SHARP and Asia-Pacific trial results supported the approval of sorafenib as the first-line targeted treatment for advanced HCC. Additionally, sorafenib exhibits anti-tumour activity in cases of recurring malignancies after liver transplantation, providing a survival advantage over the best supportive treatment. In clinical practice, sorafenib was consistently safe for both Child–Pugh A and B patients, although side effects such diarrhoea and hand–foot syndrome were linked to longer overall survival (OS) [110]. The increase in survival remains moderate, and long-term sorafenib therapy causes acquired resistance to develop quickly, limiting future benefits. Furthermore, research from several nations has shown that sorafenib is not cost-effective at present pricing, which has attracted a lot of attention and criticism. This emphasises how critical it is to treat HCC in non-surgical candidates using a unique systemic strategy [111].

Although prior initiatives, including the use of anti-viral medicines, have generally failed, approaches to adjuvant therapy have been thoroughly researched due to the high recurrence rates following hepatectomy for HCC [112]. Antiangiogenic drugs; MEK/ERK, mTOR, HGF/c-Met, and EGF/EGFR pathway inhibitors; and histone deacetylase inhibitors have all been used with sorafenib. There have been phase III trials for other drugs, including interferon, selumetinib, capecitabine, tegafur-uracil, and gemcitabine and oxaliplatin (GEMOX), but none of these combinations containing sorafenib have been successful yet [113,114]. Improved postoperative survival was anticipated due to sorafenib’s potential in the adjuvant situation based on its successful palliative use. Unfortunately, this has not been proven, and in the STORM trial and other Western trials, it did not diminish postoperative tumour recurrence [115,116]. However, sorafenib dramatically lowers tumour recurrence and improves disease-free survival (DFS) in BCLC (Barcelona Clinic Liver Cancer Classification) stage C patients, and Zhuang et al. showed that adjuvant therapy improved both DFS and OS [116]. In contrast to intermediate HCC, sorafenib treatment after hepatectomy dramatically extended the OS of advanced HCC. Regardless of the BCLC stage, patients who underwent hepatectomy and were found to have microvascular invasion (MVI) upon a pathological examination benefited from adjuvant sorafenib therapy [112,117]. Following resection, sorafenib dramatically increased overall and recurrence-free survival (RFS), according to data from a sizable recent trial with propensity score matching analysis. Sorafenib’s effect in MVI patients after radical resection was being investigated in a phase III clinical trial (NCT02867280), but the study was terminated due to negative results obtained in the midterm analysis. A study (NCT02537158) with similar objectives is active, but the information on recruiting patients is not available [118,119].

**Figure 1:**
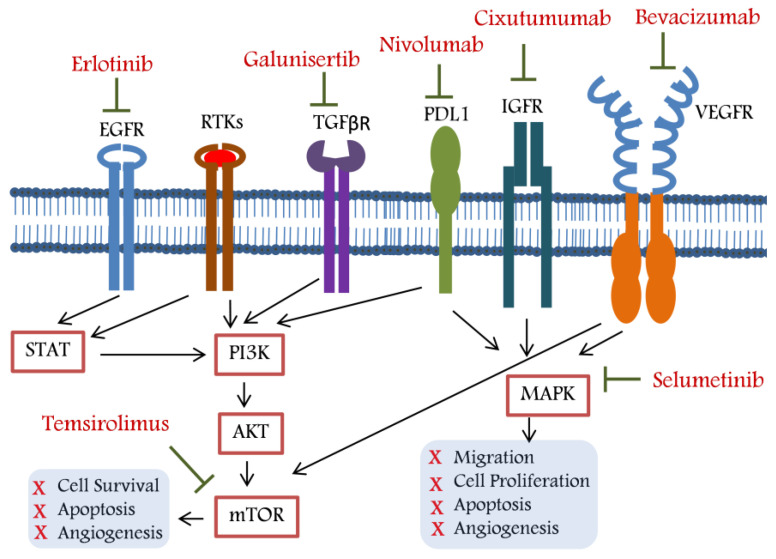
Therapeutic targets and respective drugs used for unresectable hepatocellular carcinoma. Erlotinib inhibits EGFR and thereby negatively regulates cancer hallmarks through modulating the STAT/PI3K/Akt/mTOR axis. Galunisertib inhibits TGFβR which in turn negatively regulates cancer hallmarks through the PI3K/Akt/mTOR axis. Nivolumab, cixtumumab, and bevacizumab inhibit PDL1, IGFR, and VEGFR, respectively, and thereby negatively regulate cancer hallmarks through MAPK. Temsirolimus and selumetinib directly inhibit mTOR and MAPK, respectively, and thereby negatively regulate cancer hallmarks in recurrent hepatocellular carcinoma.

Similar to early attempts to create first-line medicines that were superior to sorafenib, preliminary attempts to identify efficient second-line therapies were unsuccessful. The RESORCE trial (phase III) examined regorafenib efficacy in sorafenib-treated worsened patients. This study officially launched the second-line and sequential therapy era by proving the efficacy of second-line medicines. Regorafenib improved survival regardless of how quickly the disease had advanced during or following the last sorafenib dosage. Even in patients with HCC recurrence after liver transplantation, regorafenib was demonstrated to be successful for the sequential therapy of sorafenib followed by regorafenib. Not all patients who respond to sorafenib are, however, eligible for second-line treatment [120]. Only about 30% of patients in clinical practice are eligible for second-line regorafenib therapy [121]. The effectiveness and superior results of following treatment, including lenvatinib, were influenced by the liver’s functional reserve and ECOG (Eastern Cooperative Oncology Group) performance status during sorafenib therapy [122]. Despite these targeted medications discussed above, the search for novel agents has continued. Components of targeted therapy (targets and respective drug) for recurrent hepatocellular carcinoma are summarised in Figure 1.

## 4. Conclusions

In this review, we have discussed numerous possible therapeutic targets and associated drugs which have been studied in HCC clinical patients. We focused on the drugs studied for their effect on patient survival (disease-free and/or overall). Immune checkpoint inhibitors (ICIs), including pembrolizumab, nivolumab, durvalumab, and atezolizumab, have recently been studied in HCC patients, although clinical trials evaluating single-agent ICI have revealed unimpressive outcomes. Immune-based combinations, on the other hand, have proved more dramatic. In reality, a new standard of care for HCC patients with advanced disease has been established as a result of the phase III IMbrave150 trial, which compared sorafenib with atezolizumab–bevacizumab. The study revealed better therapeutic outcomes (in terms of survival and therapeutic response) in patients receiving the immune-based combination. Despite the fact that ICIs appear to have finally discovered their place in combinatorial treatments for HCC, there are still a number of unsolved problems. Since only a portion of HCC patients respond to immunotherapy, the lack of established biomarkers of response is one of the important problems. Based on these assumptions, it is crucial to comprehend the function of prospective biomarkers, such as the expression of the programmed death ligand 1 (PD-L1), tumour mutational burden (TMB), microsatellite instability (MSI) status, gut microbiota, and many more. Additionally, the medicines, patients, research methods, study phases, and clinical results used in clinical trials of HCC immunotherapy vary greatly.

None of the targeted therapies decreased the HCC recurrence up to the mark. Moreover, the review indicates that studying the role of targeted therapy alone or in combination is a leading field in HCC research, considering the clinical impact. Even though some of the aforementioned targets may appear promising, there are still some issues with studying this subject. For example, the recurrence rate increases with the advancement of HCC; thus, the targeted therapy should be categorised for the initial, intermediate, and advanced stages of the disease to achieve a better clinical outcome. The metastasis and angiogenesis-related targets may be targeted using appropriate drugs for patients with advanced HCC. The therapeutic drugs may be used in combination with toxicity-lowering agents, which could enhance the therapeutic potential and quality of life, and decrease recurrence in patients. Combinations of selumetinib and erlotinib; selumetinib and temsirolimus; erlotinib and galunisertib/nivolumab; and nivoluab and selumetinib/temsirolimus may be studied in HCC clinical trials, as these combination either target different molecular axes or strengthen the activities of their direct target inhibition in association with the downstream inhibitor partner (Figure 1). Slow-release formulations of the targeted therapeutic drug may be developed for initial and intermediate HCC, which may delay the recurrence process. In conclusion, more effective targeted therapy, either alone or in combination, is urgently needed to deal with the recurrence problem in HCC patients, which will help in the fight against this devastating disease.

## Figures and Tables

**Table 1:** Clinical trials on targeted therapy in hepatocellular carcinoma recurrence.

Study Design	Clinical Trila.Gov Identifier	Phase/Type	Primary End Point	Secondary End Point	Study Status
Galunisertib + Nivolumab	NCT02423343	I/II	MTD	PK/Cmin/PFS/CR/TTR/OS	Completed
Huaier Granule	NCT01770431	IV	IRMAH	PSP	Completed
Sofosbuvir + Ribavirin	NCT01559844	II	pTVR	VF	Completed
Peretinoin	NCT01640808	III	RFS	DFS/TTR	Completed
Bevacizumab	NCT00055692	II	PFS/CR + PR	-	Completed
Sorafenib	NCT00692770	III	RFS	TTR/OS	Completed
Doxorubicin + Bortezomib	NCT00083226	II	RECIST	PFS/OS	Completed
Cixutumumab	NCT00639509	II	PFS/ORR	OS	Completed
Selumetinib	NCT00604721	I	CR + PR	PFS/OS	Completed
Belinostat	NCT00321594	I/II	DLT/MTD		Completed
Bevacizumab + Erlotinib	NCT00365391	II	ORR	TTP/PFS/OS	Completed
Bevacizumab + Temsirolimus	NCT01010126	II	CR + PR/T	-	Completed
Sorafenibtosylate	NCT01502410	II	CR + PR	T/PK	Completed
Biological: -205/NY-ESO-1 Fusion Protein CDX-1401 ± Sirolimus	NCT01522820	I	-	-	Completed
Sustained released 5-FU and cisplatin	NCT00817895	Interventional	-	-	Completed
Statin therapy and the risk of HCC recurrence	NCT03490461	Observational	-	-	Completed
Ginsenoside Rg3	NCT01717066	Interventional	-	-	Completed
Lenvatinib	NCT04415567	Observational	-	-	Completed
Direct Acting Antivirals against hepatitis C virus infection	NCT03197155	Observational	-	-	Completed
Rapamycin	NCT02724332	I	-	-	Completed
Ispinesib	NCT00095992	II	-	-	Completed
Licartin	NCT00819650	II	-	-	Completed
Epirubicin and lipiodol	NCT00820053	II	-	-	Completed
Ethiodised oil	NCT00870558	III	-	-	Completed
Bortezomib	NCT00077441	II	-	-	Completed
Gefitinib	NCT00071994	II	-	-	Completed
Interferon-alfa-2b and Ribavirin	NCT00375661	IV	-	-	Completed
Cixutumumab and Sorafenib Tosylate	NCT01008566	I	-	-	Completed
Doxorubicin hydrochloride and Nolatrexed dihydrochloride	NCT00012324	III	-	-	Completed
Erlotinib hydrochloride	NCT00047346	I	-	-	Completed
Oxaliplatin	NCT00052364	II	-	-	Completed
Interferon alpha-2b	NCT00273247	III	-	-	Completed
Oxaliplatin	NCT00091182	II	-	-	Completed
Sorafenib tosylate	NCT00844168	I	-	-	Completed

Dose-limiting toxicities (DLT); maximum tolerated dose (MTD); complete response + partial response (CR + PR); median progression-free survival (PFS); median overall survival (OS); overall response rate (ORR); response evaluation criteria in solid tumours (RECIST); recurrence-free survival (RFS); time to recurrence (TTR); disease-free survival (DFS); percentage of participants with post-transplant virologic response (pTVR); virologic failure (VF); incidence of recurrence and metastasis after hepatectomy (IRMAH); postoperative survival period (PSP); maximum tolerated dose (MTD); pharmacokinetics (PK); minimum concentration (Cmin); time to progression (TTP); toxicity (t).
